# A Dexamethasone Prodrug Reduces the Renal Macrophage Response and Provides Enhanced Resolution of Established Murine Lupus Nephritis 

**DOI:** 10.1371/journal.pone.0081483

**Published:** 2013-11-28

**Authors:** Fang Yuan, Dana E. Tabor, Richard K. Nelson, Hongjiang Yuan, Yijia Zhang, Jenny Nuxoll, Kimberly K. Bynoté, Subodh M. Lele, Dong Wang, Karen A. Gould

**Affiliations:** 1 Department of Pharmaceutical Sciences, University of Nebraska Medical Center, Omaha, Nebraska, United States of America; 2 Department of Genetics, Cell Biology & Anatomy, University of Nebraska Medical Center, Omaha, Nebraska, United States of America; 3 Department of Pathology and Microbiology, University of Nebraska Medical Center, Omaha, Nebraska, United States of America; Beth Israel Deaconess Medical Center, United States of America

## Abstract

We evaluated the ability of a macromolecular prodrug of dexamethasone (P-Dex) to treat lupus nephritis in (NZB × NZW)F1 mice. We also explored the mechanism underlying the anti-inflammatory effects of this prodrug. P-Dex eliminated albuminuria in most (NZB × NZW)F1 mice. Furthermore, P-Dex reduced the incidence of severe nephritis and extended lifespan in these mice. P-Dex treatment also prevented the development of lupus-associated hypertension and vasculitis. Although P-Dex did not reduce serum levels of anti-dsDNA antibodies or glomerular immune complexes, P-Dex reduced macrophage recruitment to the kidney and attenuated tubulointerstitial injury. In contrast to what was observed with free dexamethasone, P-Dex did not induce any deterioration of bone quality. However, P-Dex did lead to reduced peripheral white blood cell counts and adrenal gland atrophy. These results suggest that P-Dex is more effective and less toxic than free dexamethasone for the treatment of lupus nephritis in (NZB × NZW)F1 mice. Furthermore, the data suggest that P-Dex may treat nephritis by attenuating the renal inflammatory response to immune complexes, leading to decreased immune cell infiltration and diminished renal inflammation and injury.

## Introduction

Lupus nephritis is a leading cause of morbidity and mortality among lupus patients [1]. Lupus nephritis is associated with inflammation caused by renal deposition of immune complexes containing autoantibodies, particularly IgG autoantibodies recognizing double stranded DNA (anti-dsDNA IgG). If not resolved, renal inflammation can lead to renal injury, dysfunction, and failure. 

Lupus nephritis can be effectively treated with glucocorticoids (GCs). However, because long-term GC therapy is required, this treatment frequently is associated with numerous side effects involving the endocrine, cardiovascular, hematopoietic and musculoskeletal systems [2]. These adverse side effects, especially secondary osteoporosis, contribute significantly to morbidity in lupus patients. Nevertheless, because of the lack of alternative therapeutic options, GCs continue to be the mainstay of clinical management of lupus nephritis [3]. 

In an attempt to reduce GC-associated side effects, we previously employed a nanomedicine-based strategy to modify the pharmacokinetic/biodistribution profile of GCs to enhance drug delivery to the site of inflammation while reducing systemic exposure to the drug. Specifically, we developed a macromolecular prodrug of dexamethasone (P-Dex); P-Dex is taken up preferentially by the proximal tubule epithelial cells in the inflamed kidneys of (NZB × NZW)F1 females, but the prodrug is also found to a much lesser extent in splenocytes and circulating blood cells [4]. We observed that P-Dex prevents the development of nephritis in young lupus-prone (NZB × NZW)F1 female mice without causing osteoporosis, a side effect associated with the equivalent dose of free Dex [4]. Our previous studies also suggest that P-Dex prevents nephritis by attenuating the response of the kidney to immune complex deposition and decreasing the recruitment of infiltrating immune cells to the kidney. 

Here, we sought to further explore the therapeutic potential of P-Dex for the treatment of lupus nephritis using a preclinical mouse model. The primary objective of the present study was to determine if P-Dex could effectively treat established nephritis in (NZB × NZW)F1 mice. Additionally, we sought to assess the safety of longer term P-Dex administration and to further explore the potential underlying mechanism of action of this prodrug. 

## Results

### P-Dex reverses established albuminuria, extends survival and reduces incidence of severe nephritis and tubulointerstitial disease in (NZB × NZW)F1 mice

**Figure 1:**
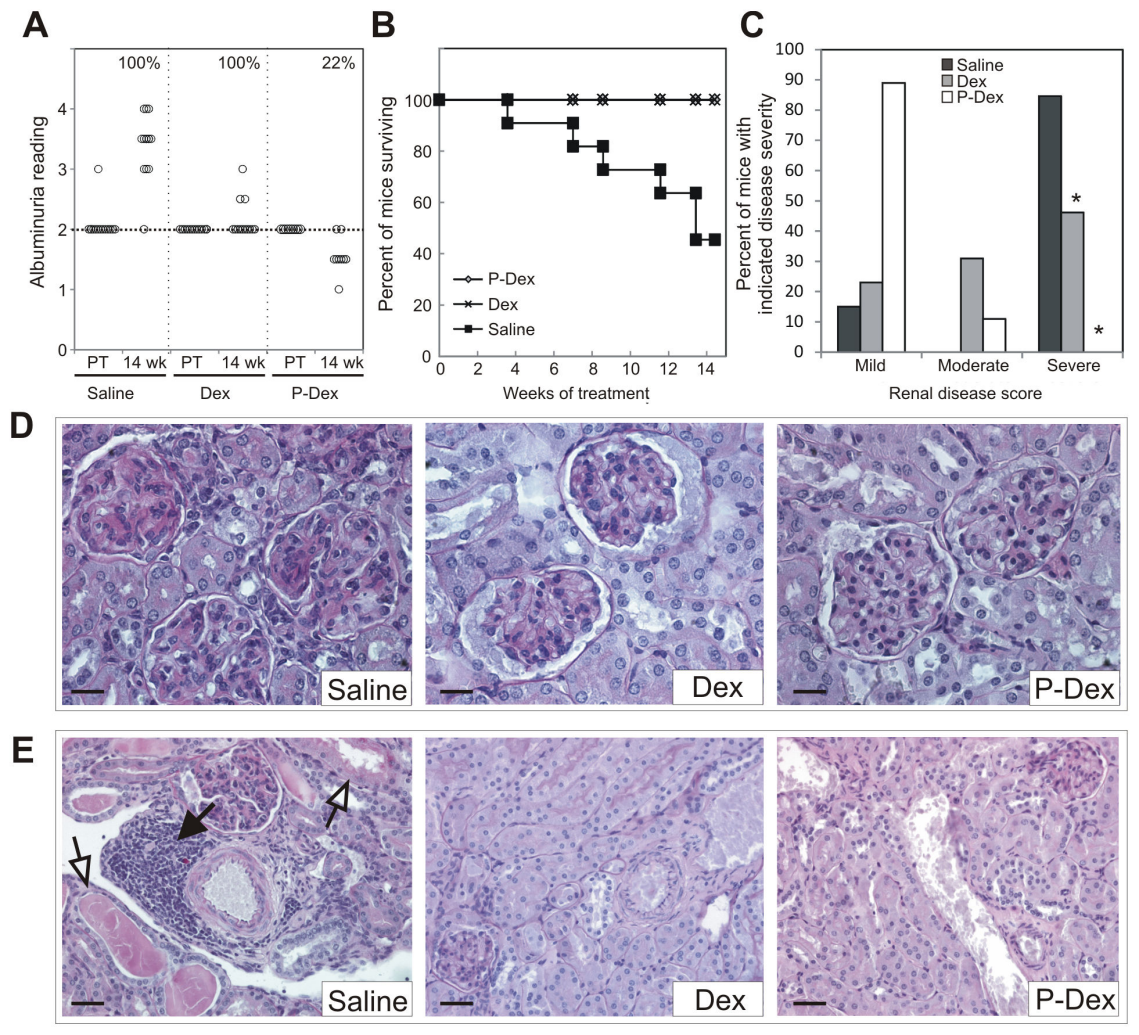
P-Dex ameliorates albuminuria, extends lifespan and attenuates development of severe nephritis and tubulointerstitial disease in (NZB × NZW)F1 females. (A), Albuminuria data for mice in saline (n=13), Dex (n=13), and P-Dex (n=9) treatment groups is illustrated at the pretreatment (PT) and 14-week time points. The incidence of albuminuria at the 14-week time point for each group is shown (in %) in upper right corner of each sub-section. For mice in the saline group that did not survive to the 14-week time point (n=7), the albuminuria reading shown is the last recorded value. (B), A Kaplan-Meier survival curve for each treatment group is shown. (C), The fraction of mice in each treatment group with mild, moderate and severe renal disease is shown. (D), A PAS stained histological section illustrating representative glomeruli from each treatment group are provided. Scale bars: 20 μm. (E), A representative PAS stained histological section illustrating the tubulointerstitium from each treatment group is provided. Scale bars: 40 μm. The asterisk (*) indicates a statistically significant difference (*P* < 0.05) from the saline control group.

To determine if P-Dex could ameliorate established nephritis, P-Dex was administered monthly to (NZB × NZW)F1 females beginning at ~22 weeks of age, after they had developed nephritis, as evidenced by sustained albuminuria. Treatment was continued for 12 weeks. Two control groups, one receiving dose equivalent daily Dex and the other receiving a monthly dose of saline, were also treated for 12 weeks. Mice were monitored for an additional two weeks after cessation of treatment. Over the entire experimental time course, albuminuria not only persisted in 100% of the mice in the saline treated group, but also increased in severity in most of these mice (93%) (Figure 1A). In the Dex group, albuminuria likewise continued in 100% of the mice. However, albuminuria intensified in just 23% of the Dex treated mice, indicating that Dex treatment could prevent progression of renal dysfunction. By contrast, albuminuria resolved in 78% of the mice in the P-Dex group (Figure 1A). Albuminuria persisted but did not increase in the remaining 22% of mice in this group. The fraction of mice in the P-Dex group that showed resolution of albuminuria was significantly greater than that in the Dex treated group, indicating that P-Dex is more effective than dose equivalent Dex in resolving albuminuria associated with lupus nephritis (*P* ≤ 1x10^-6^).

Prior to the end of the experiment, ~55% of mice in the saline group were euthanized due to severe nephritis (Figure 1B). In the saline treated group, median survival was ~13 weeks after initiation of treatment, which corresponded to ~35 weeks of age. The median survival in this group is similar to what we and others have reported previously for (NZB × NZW)F1 females [5-7]. All mice in the Dex and P-Dex groups survived the entire treatment period, indicating that both therapies significantly increased the fraction of mice surviving until the end of the treatment period (*P* = 0.001). These data indicate that Dex and P-Dex can extend the lifespan of (NZB × NZW)F1 mice. 

The kidneys from 86% of mice in the saline group showed histological evidence of severe glomerulonephritis, characterized by diffuse glomerular hypercellularity, matrix deposition and crescent formation (Figure 1 C, D). By contrast, in the Dex treated group, the incidence of severe glomerulonephritis was 46%, which was significantly less than that in saline controls (Figure 1C, D; P = 0.04). In the P-Dex treated group, none (0%) of the kidneys showed histological evidence of severe glomerulonephritis; incidence of severe nephritis in this group was different than that in the saline and Dex groups (*P* <1 x10^-3^). 

Furthermore, 100% of the kidneys from mice in the saline group showed evidence of marked tubulointerstitial disease, which was typified by tubular dilation, tubular casts, and immune cell infiltration into the interstitium. The immune cell infiltrates were found in both the cortex and medulla, and were distributed throughout the interstitium as well as in prominent perivascular lymphoid aggregates (Figure 1E). By contrast, in both the Dex and P-Dex groups, there was little indication of tubulointerstitial disease; kidneys displayed mild tubular dilation, sparse tubular casts and scant evidence of interstitial immune cell infiltration (Figure 1E).

### P-Dex does not reduce serum anti-dsDNA IgG or glomerular immune complexes

**Figure 2:**
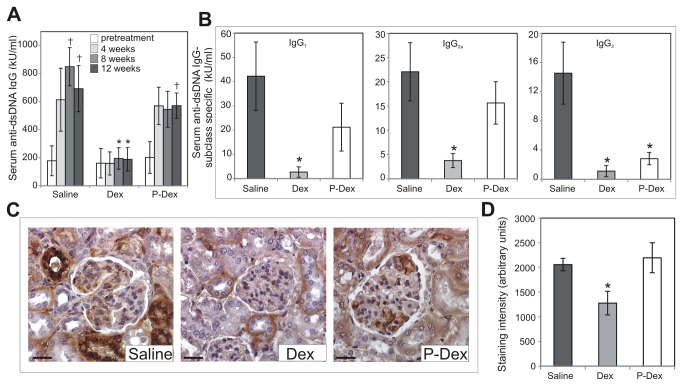
The effect of treatment on serum anti-dsDNA IgG and renal immune complexes. (A), Anti-dsDNA IgG levels for mice in saline (n=13), Dex (n=13), and P-Dex (n=9) treatment groups were determined via ELISA at pretreatment, 4-week, 8-week, and 12 week time points. For the saline group, serum was available for analysis only from the subset of mice surviving at each time point: 12 mice at 4-week time point; 11 mice at 8-week time point; 9 mice at 12-week time point (B), Levels of anti-dsDNA IgG of each subclass were determined via ELISA at the 12-week time point. For the saline group, serum was available for this analysis only from the 9 mice that survived to the 12-week time point (C), Representative sections of kidney from each treatment group are shown. Sections were stained for renal deposition of anti-dsDNA IgG via immunohistochemistry. (D), Quantification of immune complex staining is illustrated. Scale bars: 25 μm; The asterisk (*) indicates a statistically significant difference (*P* < 0.05) from the saline control group. The dagger (†) indicates a statistically significant difference (*P* < 0.05) from the pretreatment time point of the same treatment group.

In (NZB × NZW)F1 mice, severity of nephritis typically correlates with serum levels of pathogenic autoantibodies, particularly anti-dsDNA IgG [8]. Therefore, serum anti-dsDNA IgG levels were assessed. In the saline group, serum anti-dsDNA IgG levels increased significantly over the experimental time course (Figure 2A; P = 0.02). By contrast, in the Dex group, serum anti-dsDNA IgG did not change significantly over the experimental time course (Figure 2A; P > 0.05). Consequently, at the end of the experimental time course, serum anti-dsDNA IgG levels in the Dex group were significantly lower than those in the saline group (*P* = 0.02). Serum anti-dsDNA IgG levels increased in the P-Dex group over the experimental time course (Figure 2A; P = 0.03). There were no significant differences in serum anti-dsDNA IgG levels between the P-Dex and saline groups (*P* > 0.05). However, serum anti-dsDNA IgG levels in the P-Dex group were significantly greater than those in the Dex group at the end of the experimental time course (*P* < 0.008). These results demonstrate that P-Dex attenuates nephritis through a mechanism independent of the production of pathogenic autoantibodies.

Although our results indicate that P-Dex did not impact the total levels of serum anti-dsDNA IgG, this observation does not preclude the possibility that P-Dex alters the relative abundance of different subclasses of anti-dsDNA IgG autoantibodies. Such an effect could be important given the fact that anti-dsDNA IgG autoantibodies of different subclasses are not equally pathogenic [8-10]. Therefore, we examined the impact of treatment on the levels of anti-dsDNA IgG of each subclass individually. In the Dex treated group, serum levels of anti-dsDNA IgG_1_, IgG_2a_, IgG_3_ and were significantly lower than in the saline control group (Figure 2B; P < 0.05). By contrast, the levels of serum anti-dsDNA IgG_1_ and IgG_2a_ autoantibodies did not differ between the P-Dex and saline groups (Figure 2B; P > 0.05). However, the P-Dex treated group did display lower serum levels of anti-dsDNA IgG_3_ autoantibodies compared to the saline group (Figure 2B; P = 0.03). There were no differences in serum levels of anti-dsDNA IgG_2b_ autoantibodies between any of the groups (data not shown). These results indicate that P-Dex does not cause a dramatic shift in the relative abundance of different subclasses of anti-dsDNA IgG. Importantly, these results also clearly illustrate that P-Dex does not decrease the abundance of anti-dsDNA IgG_2a_ autoantibodies, which are considered to be the most pathogenic autoantibodies in (NZB x NZW)F1 mice [8-10]. 

Because glomerular deposition of anti-dsDNA IgG-containing immune complexes contributes to the development and progression of nephritis [11], we evaluated the impact of treatment on glomerular immune complex deposition. In the kidneys of mice in the saline group, prominent glomerular immune complex deposition was observed (Figure 2C, D). In the kidneys of the Dex group, glomerular immune complex deposition was significantly less than that in the saline group (Figure 2C, D; *P* = 0.04). Glomerular immune complex staining in the P-Dex group was similar to that in the saline group (Figure 2C, D; *P* > 0.05), but was significantly different than that in the Dex group (*P* ≤ 0.025). Thus, P-Dex treated mice do not develop nephritis despite the presence of abundant glomerular immune complexes. 

### P-Dex reduces renal macrophage infiltration and tubulointerstitial injury

**Figure 3:**
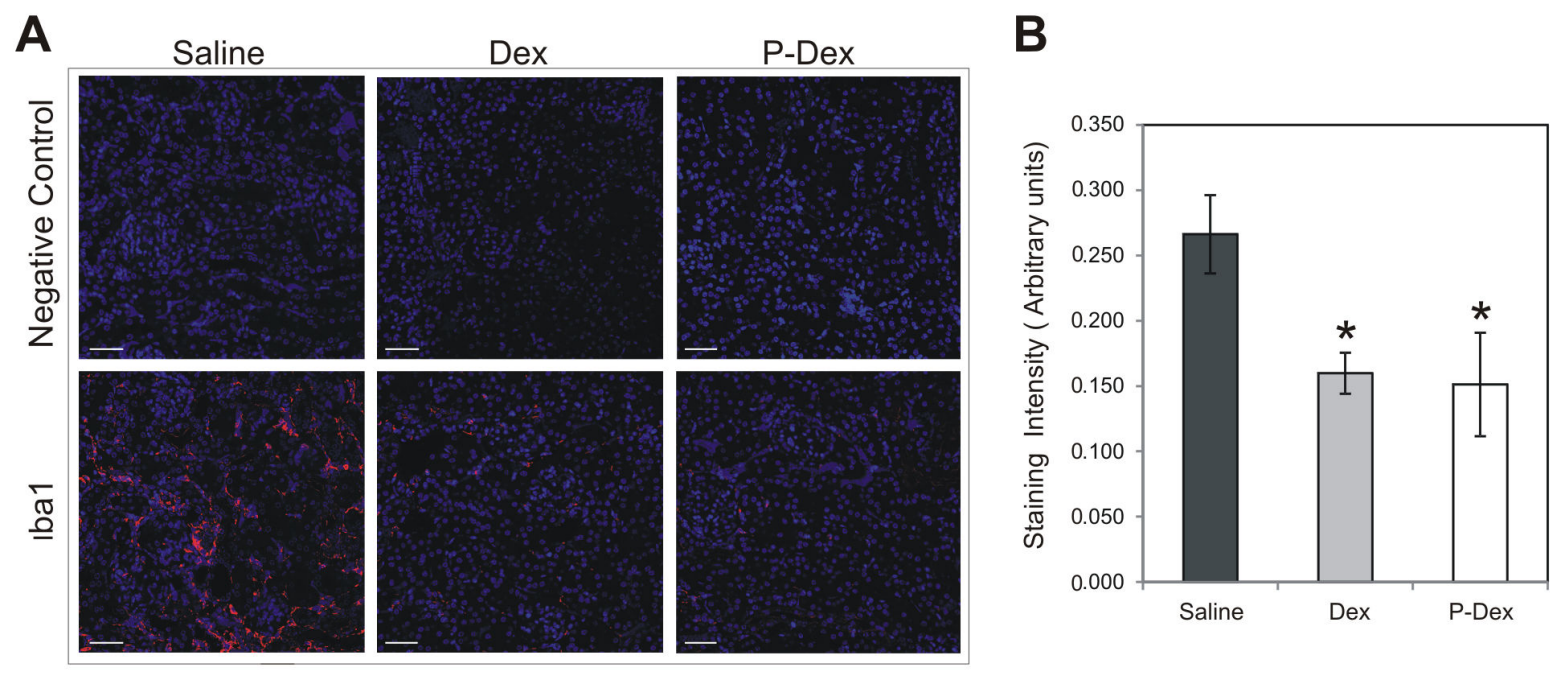
Impact of treatment on renal macrophage infiltration in (NZB × NZW)F1 mice. (A), Representative confocal images of immunohistochemical staining of kidney sections from mice in the saline (n=13), Dex (n=13), and P-Dex (n=9) treatment groups are shown. Sections were stained with an anti-Iba1 antibody (red) and DAPI (blue). Negative control (no Iba1 antibody) and merged images are shown. (B), Quantification of Iba1 staining is illustrated. Scale bars: 50 μm; the asterisk (*) indicates a statistically significant difference (*P* < 0.05) from the saline control group.

In (NZB × NZW)F1 mice, the presence of glomerular immune complexes is not sufficient for the development of nephritis. Rather, the development of nephritis requires the recruitment of FcR-expressing myeloid cells, including macrophages, to the kidney and the subsequent activation of these cells by glomerular immune complexes [12,13]. These activated macrophages are thought to contribute to the chronic renal inflammation and tissue damage associated with nephritis. Therefore, the impact of P-Dex treatment on macrophage recruitment in the kidney was evaluated. Quantification of staining with the macrophage marker Iba1 revealed abundant macrophage infiltration into the tubulointerstitium and periglomerular area in the saline group (Figure 3A,B). By contrast, macrophage infiltration in both the Dex and P-Dex groups was significantly less than that the saline control group (Figure 3A, B; *P* < 0.04). The modest recruitment of macrophages to kidneys from the P-Dex treated mice, despite the presence of abundant glomerular immune complexes, suggests that P-Dex attenuates nephritis by impairing the macrophage infiltration that occurs in response to renal immune complex deposition. 

**Figure 4:**
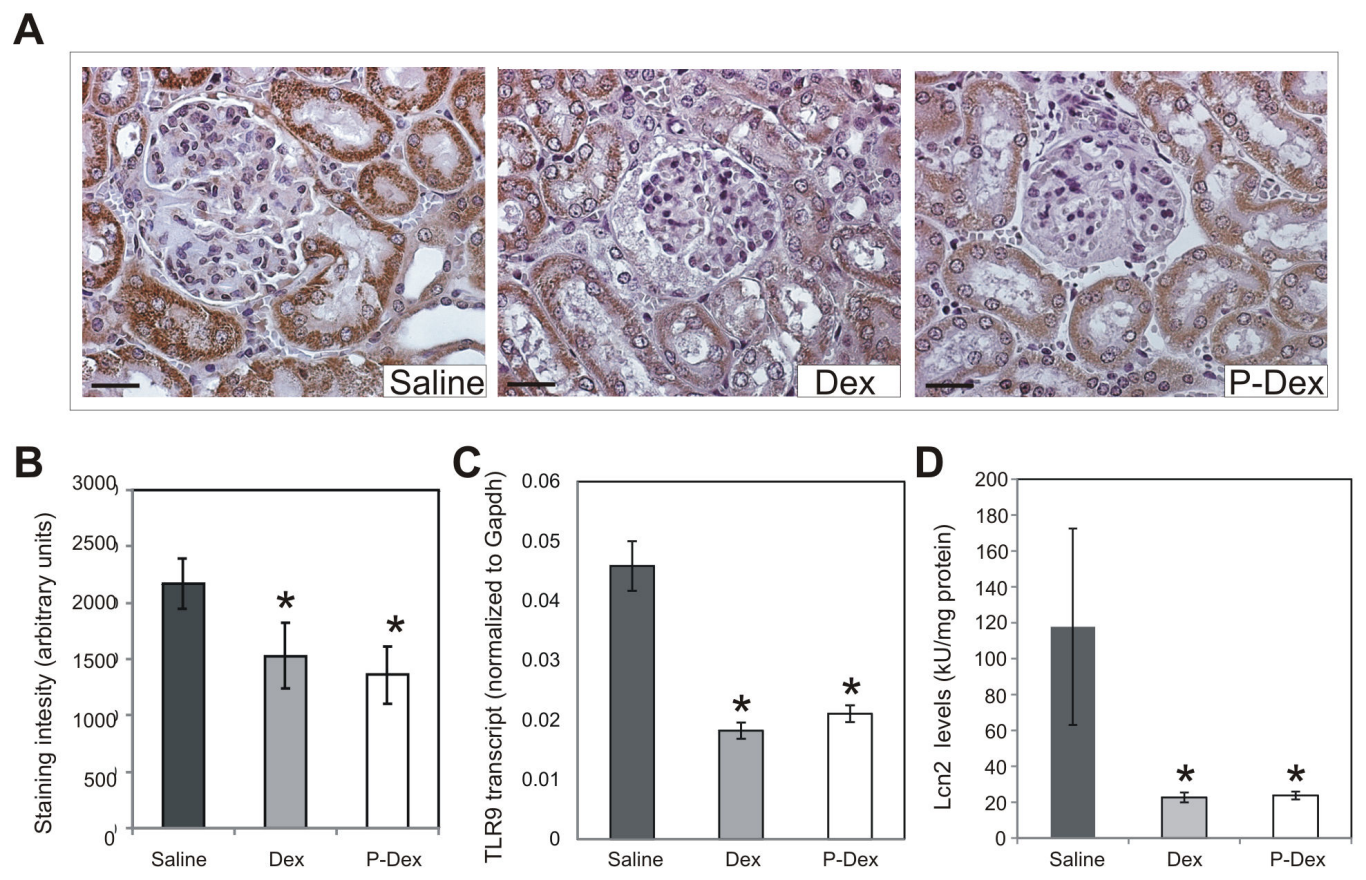
Impact of treatment on tubulointerstitial inflammation and injury in (NZB × NZW)F1 mice. (A), Representative images of immunohistochemical staining of kidney sections from mice in the saline (n=13), Dex (n=13), and P-Dex (n=9) treatment groups are shown. Sections were stained with an anti-TLR9 antibody (brown) and counterstained with hematoxylin (blue). (B), Quantification of TLR9 staining is illustrated. (C), TLR9 transcript levels in the kidney were measured by quantitative RT-PCR. For the saline group, frozen kidneys were available for RNA extraction only from the 6 mice that survived until the 14-week time point. (D), Levels of LCN2 were measured in kidney lysates by ELISA. For the saline group, frozen kidneys were available for preparation of protein lysates only from the 6 mice that survived until the 14-week time point. Scale bars: 25 μm; the asterisk (*) indicates a statistically significant difference (*P* < 0.05) from the saline control group.

The recruitment of macrophages to the kidney in lupus-prone mice leads to tubulointerstitial inflammation and injury [14,15]. To determine whether the reduced macrophage recruitment in the P-Dex group was also associated with decreased tubulointerstitial injury, the expression of Toll-like receptor 9 (TLR9) and Lipocalin 2 (LCN2), markers for renal tubule damage and tubulointerstitial injury, were assessed [16-20]. Consistent with the observation that the kidneys of the mice in the saline group contained numerous macrophages and showed pronounced tubulointerstitial disease, abundant tubular TLR9 staining was observed in this group (Figure 4A, B). By contrast, in both the Dex and P-Dex groups, there was significantly less tubular TLR9 staining than in the saline group (Figure 4A,B; *P* < 0.05). To assess the impact of Dex and P-Dex on TLR9 expression in a more quantitative fashion, quantitative RT-PCR was performed to determine the level of expression of the *Tlr9* transcript. This analysis confirmed that the levels of *Tlr9* transcript were significantly less in the Dex and P-Dex groups compared to that in the saline control group (Figure 4C; P ≤ 0.01). Likewise, there was robust expression of LCN2 in the kidneys of the saline group, and significantly less LCN2 in the kidneys of both the Dex and P-Dex groups (Figure 4D; P ≤ 0.05).

### P-Dex prevents the development of lupus-associated hypertension, splenomegaly and vasculopathy

**Figure 5:**
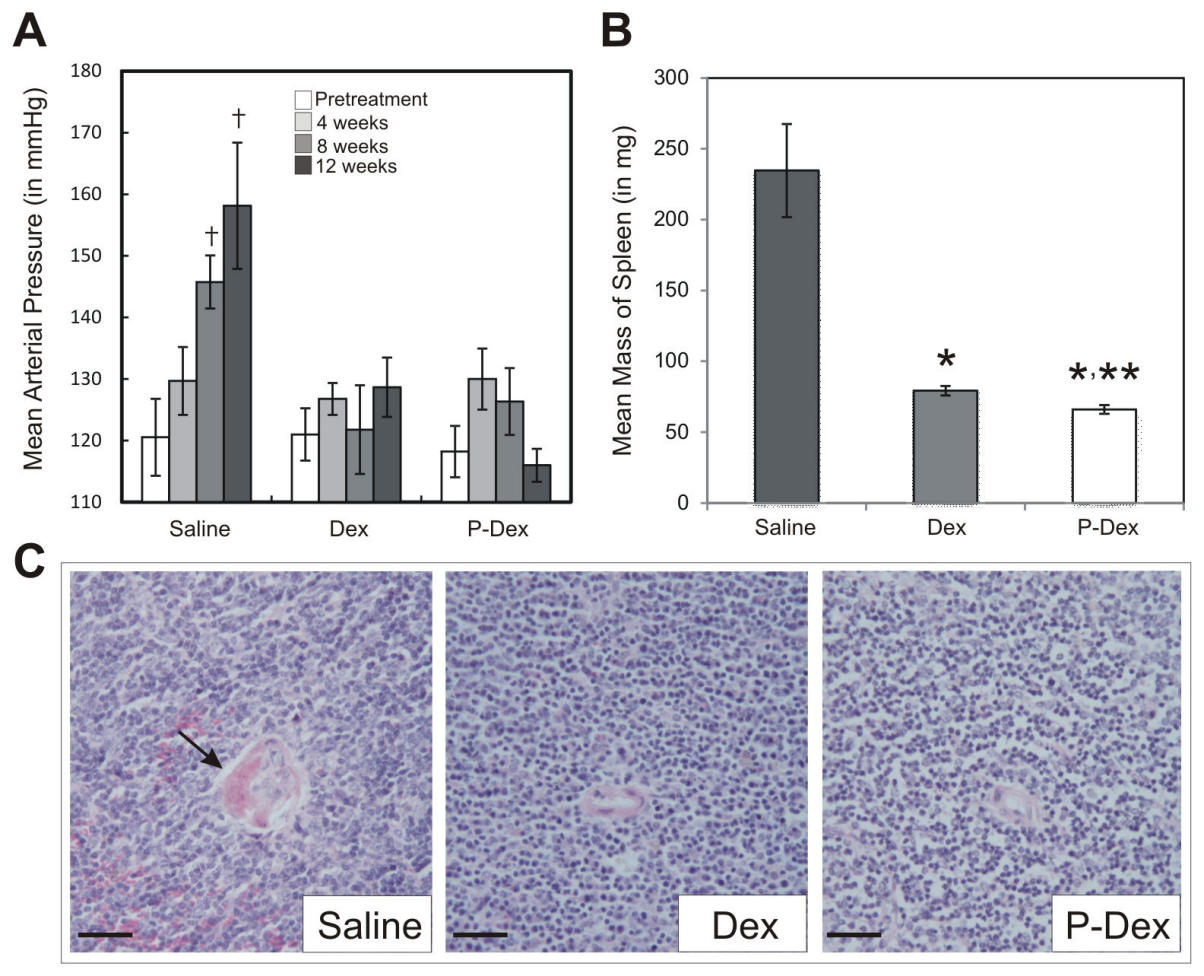
Impact of treatment on hypertension, splenomegaly and vasculitis in (NZB × NZW)F1 mice. (A), Mean arterial pressure was measured at pretreatment, 4-week, 8-week, 12 week time points via tail-cuff method. For the saline group, measurements were obtained only for the subset of mice surviving at each time point: 12 mice at 4-week time point; 11 mice at 8-week time point; 9 mice at 12-week time point (B), Spleen mass was determined at the time of sacrifice in each mouse. (C), A representative hematoxylin and eosin stained histological section illustrating a splenic vessel from each treatment group is provided. The arrow indicates perivascular fibrin deposits indicative of vasculitis. Scale bars: 50 μm; the asterisk (*) indicates a statistically significant difference (*P* < 0.05) from the saline control group. The double asterisk (**) indicates a statistically significant difference (*P* < 0.05) from the Dex group. The dagger (†) indicates a statistically significant difference (*P* < 0.05) from the pretreatment time point of the same treatment group. For saline and Dex treatments, n=13; for P-Dex treatment, n=9.

Since systemic inflammation and renal dysfunction promote hypertension in lupus patients and (NZB × NZW)F1 mice [21,22], we assessed the impact of treatment on mean arterial pressure (MAP). Prior to treatment, mice in the saline group were normotensive (Figure 4A). However, over the experimental time course, MAP rose significantly and virtually all of the mice in this group became hypertensive (Figure 5A; P ≤ 0.01). By contrast, there was no significant change in MAP in either the Dex or P-Dex groups over this time course (Figure 5A; P > 0.05). 

Splenomegaly occurs only in a subset of human lupus patients. By contrast, splenomegaly is observed in virtually all lupus prone mouse strains, including the (NZB × NZW)F1 hybrid. Therefore, spleen mass of the animals was investigated at necropsy. Splenomegaly was pronounced in the saline group (Figure 5B). By contrast, there was little evidence of splenomegaly in either the Dex or P-Dex treated groups; mean spleen mass in the Dex and P-Dex groups was significantly different than that in the saline group (*P* < 0.001). The spleen mass in the P-Dex group was significantly different than that in the Dex group (*P* = 0.03), although the biological significance of this difference is unclear. All differences persisted when spleen mass was normalized to total body mass (data not shown). Thus, both Dex and P-Dex can attenuate splenomegaly in (NZB × NZW)F1 mice.

Lupus patients are also at high risk for vasculitis. Vasculitis, evidenced by fibrinoid necrosis in the walls of the splenic blood vessels, was found in 54% of mice from the saline group. Fibrin deposits were also noted within the lumen of splenic vessels in 39% of the mice in the saline group (Figure 5C). No fibrin deposition or fibrinoid necrosis was observed in mice from the Dex and P-Dex groups, indicating that both treatments attenuated vascular disease in lupus prone mice.

### P-Dex treatment does not affect bone quality

**Figure 6:**
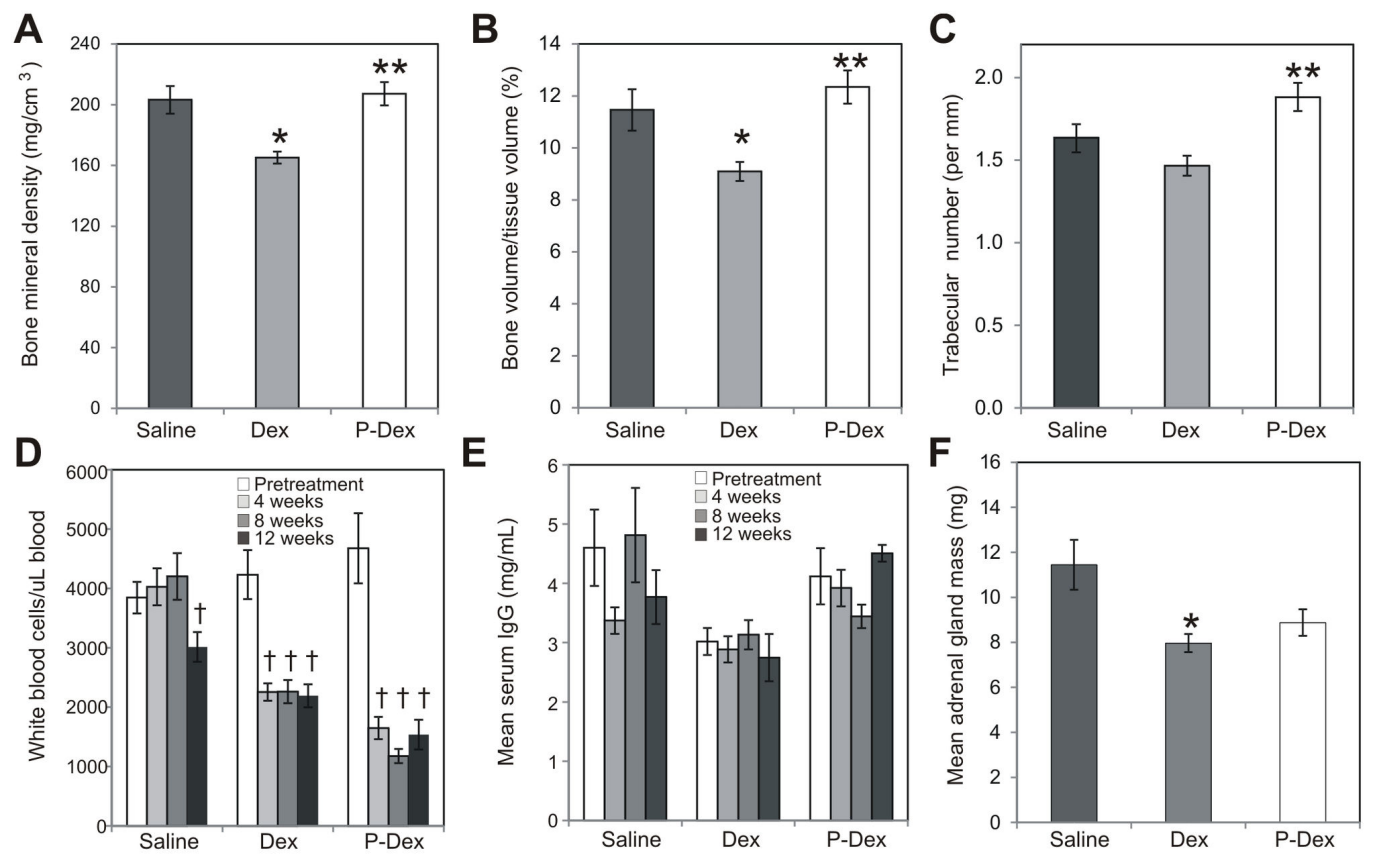
Evaluation of treatment-induced side effects. **At sacrifice, femurs were collected for endpoint analysis of bone quality**. (A), bone mineral density (B), bone volume/tissue volume and (C), trabecular number measurements in each treatment group are shown. (D), white blood cell counts and (E), total serum IgG levels were determined at pretreatment 4-week, 8-week and 12-week time points. For the saline group, measurements were obtained only for the subset of mice surviving at each time point: 12 mice at 4-week time point; 11 mice at 8-week time point; 9 mice at 12-week time point (F), Adrenal mass was determined at the time of sacrifice in each mouse. The asterisk (*) indicates a statistically significant difference (*P* < 0.05) from the saline control group. The double asterisk (**) indicates a statistically significant difference (*P* < 0.05) from the Dex group. The dagger (†) indicates a statistically significant difference (*P* < 0.05) from the pretreatment time point of the same treatment group. For saline and Dex treatments, n=13; for P-Dex treatment, n=9.

Osteoporosis is a major adverse side effect of long-term use of GCs [23]. To investigate the impact of P-Dex on the skeleton, the femoral BMD and micro-architecture were evaluated. As expected, the mean bone mineral density (BMD) and trabecular bone volume/tissue volume (BV/TV) in the femurs of Dex treated mice were significantly lower than that observed in the saline group (Figure 6A, B; *P* < 0.05). Trabecular number did not differ significantly between the Dex and saline groups (Figure 6C; P > 0.05). In the P-Dex group, mean femoral BMD, BV/TV and trabecular number did not differ significantly from the means in the saline group (Figure 6A, B, C; P > 0.05). By contrast, compared to the Dex group, the P-Dex group exhibited significantly greater BMD (Figure 6A; P = 0.004), BV/TV (Figure 6B; P = 0.007) and trabecular number (Figure 6C; P = 0.01). Thus, unlike Dex, P-Dex did not negatively affect BMD or microarchitecture of the bone.

### P-Dex treatment reduces peripheral white blood cells but does not reduce serum IgG levels

GC therapy is associated with immunosuppression [24,25]. Therefore, we monitored peripheral white blood cell (WBC) counts and serum IgG levels during the experimental time course. Prior to treatment, no significant differences in peripheral WBC counts were observed between groups (Figure 6D; P > 0.05). In the saline group, peripheral WBC counts initially remained constant, but were significantly reduced at the 12 week time point (Figure 6D; P = 0.049). By contrast, peripheral WBC counts were significantly reduced by the 4-week time point in the Dex (Figure 6D; P = 0.0004) and P-Dex (Figure 6D; P = 0.0007) groups; WBC counts remained low in both of these treatment groups for the duration of the study. No significant changes in serum IgG levels were observed in any group (Figure 6E; P > 0.05). Thus, treatment with either Dex or P-Dex reduced peripheral WBC counts, but did not reduce serum IgG levels.

### P-Dex treatment induces adrenal gland atrophy

GC therapy causes suppression of hypothalamic-pituitary-adrenal (HPA) axis and atrophy of the adrenal glands. Therefore, at necropsy, we determined the mass of the adrenal glands in each mouse. The mean adrenal mass in the Dex group was significantly different than the saline group (Figure 6F; P = 0.01). Although the mean adrenal mass in the P-Dex group was less than that in the saline group, this difference fell short of statistical significance (Figure 6F; P = 0.07). There was no significant difference in adrenal gland mass between the P-Dex and Dex groups (Figure 6F; P >0.05). These data suggest that treatment with either Dex or P-Dex induced adrenal gland atrophy. 

## Discussion

Nanomedicine-based approaches that permit the modulation of the *in vivo* pharmacokinetic/biodistribution profile of drugs represent a promising strategy for the development of novel therapeutics to treat lupus nephritis. This approach is particularly helpful for existing drugs, such as glucocorticoids, for which high potency is accompanied by severe side effects due to ubiquitous distribution within the body. 

Previously, we demonstrated that P-Dex can be passively targeted to the kidneys of lupus-prone mice, likely due to the leaky vasculature in this inflamed tissue. In the kidneys of lupus-prone mice, P-Dex is internalized and activated by proximal tubule epithelial cells. In our previous study, we found that P-Dex is more effective than free Dex at preventing nephritis in young (NZB × NZW)F1 mice, but it does not cause typical Dex-associated side effects, such as osteopenia [4]. 

In the present study, we evaluated the potential of P-Dex to treat established nephritis in (NZB × NZW)F1 mice. Over the experimental time course, albuminuria intensified in the mice in the saline control group, and almost all of these mice developed severe nephritis. Furthermore, 55% of the saline treated mice succumbed to severe nephritis. By contrast, all the animals treated with either Dex or P-Dex survived the entire 14-week experimental time course. Although albuminuria worsened in only a fraction of the mice in the Dex group, albuminuria persisted in all of the mice in this group. Strikingly, albuminuria was eliminated in almost 80% of the mice in the P-Dex group. These data indicate the P-Dex improved renal function whereas the equivalent dose of free Dex only maintained the extant level of renal function. 

Consistent with our previous observations, we found that P-Dex did not attenuate nephritis by reducing serum anti-dsDNA IgG or glomerular immune complex deposition. We also determined that P-Dex did not attenuate nephritis by causing a shift toward less pathogenic subclasses of anti-dsDNA IgG. However, P-Dex did reduce the infiltration of macrophages into the kidney. The modest recruitment of macrophages to the kidney in the P-Dex group, despite the presence of abundant glomerular immune complexes, suggests that P-Dex may impair the renal pro-inflammatory response to immune complex deposition. It has been suggested that stimulation of the TLR and FcR signaling pathways may act synergistically to initiate the chronic inflammation that leads to nephritis [26]. Both of these pathways are activated by the presence of immune complexes containing dsDNA. Our observation that P-Dex inhibits both tubulointerstitial TLR9 expression as well as the recruitment of FcR-bearing macrophages to the tubulointerstitium, despite the presence of such immune complexes, suggests that the ability to inhibit both of these pathways may contribute to the enhanced therapeutic benefit of P-Dex.

P-Dex also attenuated tubulointerstitial injury and disease. In lupus patients, tubulointerstitial inflammation and injury correlate with impaired renal function more strongly than glomerular damage [27]. Furthermore, tubulointerstitial inflammation is the best predictor of risk of progression to renal failure in lupus nephritis patients [27,28]. Altogether, these data suggest that P-Dex may restore renal function and extend lifespan in (NZB × NZW)F1 females by reducing inflammatory cell infiltration in the kidney, thereby minimizing tubulointerstitial inflammation and protecting the tubulointerstitium from injury. By contrast, Dex inhibits nephritis and extends lifespan in lupus prone mice by reducing serum levels of pathogenic anti-dsDNA IgG autoantibodies. 

In our previous study, we found that P-Dex was taken up not only by cells in the kidney, but also by spleen cells in (NZB × NZW)F1 mice [4]. Therefore, in the present study, we evaluated the impact of treatment on the spleen. Neither Dex nor P-Dex induced histopathological abnormalities in spleen. Rather, both treatments attenuated the splenomegaly that develops in (NZB × NZW)F1 mice. Dex and P-Dex also prevented the development of vasculitis affecting the splenic blood vessels. 

Due to the passive-targeting of P-Dex to the inflamed kidney, one would expect P-Dex to exhibit a superior safety profile compared to free Dex. To assess the side effects of P-Dex, we measured femoral bone quality, serum IgG levels, peripheral WBC counts and adrenal gland mass. All of these parameters are usually reflective of GC-associated toxicities (e.g. osteoporosis, adrenal gland atrophy and immunosuppression). As expected, Dex treatment significantly reduced femoral BMD and other micro-architecture parameters. By contrast, femoral BMD, trabecular BV/TV and trabecular number in the P-Dex group was not different than that in the saline group. Mice in the Dex and P-Dex groups displayed similar reductions in peripheral WBC counts and adrenal gland atrophy. This residual toxicity in the P-Dex group is likely due to the fact that P-Dex is taken up to some degree by splenocytes and circulating blood cells [4]. Additionally, these side effects could be due to the free dexamethasone that is released as a result of the cleavage of P-Dex. Collectively, these data suggest that P-Dex treatment partially eliminates the side effects associated with free Dex treatment. Further study is needed to understand why the skeleton was shielded from GC-associated toxicity following P-Dex treatment whereas other tissues and organs remained vulnerable. Acquisition of such knowledge may provide insight that would facilitate the further optimization of the design of this prodrug to improve its safety profile. 

## Materials and Methods

### Ethics statement

Mice were housed under controlled humidity, temperature and lighting conditions in facilities accredited by the American Association for Accreditation of Laboratory Animal Care, operating in accordance with standards set by the Guide for the Care and Use of Laboratory Animals (The National Academies Press, 1996). Mice were given Harlan irradiated rodent diet 7904 (Harlan Teklad, Madison, WI) and allowed to feed *ad libitum*. All procedures involving live animals were approved by the University of Nebraska Medical Center Institutional Animal Care and Use Committee under protocol 03-008. 

### Experimental animals and drug treatment

Beginning at 20 weeks of age, (NZB × NZW)F1 female mice (Jackson Laboratories, Bar Harbor, ME) groups of mice were randomized into saline, Dex and P-Dex groups and monitored weekly for albuminuria using Albustix (Siemens Corp., Washington DC). Albustix readings between 1 and 2 (30-99mg/dl) are considered “normal”, whereas readings of ≥2+ (≥100mg/dl) indicate the presence of albuminuria. Only the mice in each group with established nephritis, evidenced by sustained albuminuria (≥100 mg/dl) over an initial monitoring period of 3 weeks, were officially enrolled in the study and are described here. The P-Dex (250 mg/kg, containing 30 mg/kg of dexamethasone) and saline groups were administered monthly i.v. injections. The third group was given daily i.p. injections of dexamethasone 21-phosphate disodium (Dex, 1.32 mg/kg, containing 1.00 mg/kg of dexamethasone, Hawkins, Inc., Minneapolis, MN). Over the three-month (12 weeks) treatment period, the overall dose of dexamethasone in the P-Dex and Dex groups was the same. P-Dex was synthesized as described previously [4,29].

Every month, serum was isolated from peripheral blood, mean arterial pressure was recorded via tail-cuff method using the CODA blood pressure measuring system and software (Kent Scientific, Torrington, CT), and peripheral white blood cells were isolated from whole blood and counted by hemocytometer. Mice were weighed and monitored for albuminuria on a weekly basis. Visual inspection of mice showing evidence of increasing albuminuria and/or weight loss was performed daily. Mice that developed severe albuminuria (≥ 2000 mg/dl) or showed signs of distress (i.e. reduced mobility, weight loss >20%, edema, unkempt appearance) were sacrificed immediately. The remaining mice were sacrificed two weeks after cessation of treatment (14 weeks after initiation of treatment). All mice were sacrificed by CO_2_ asphyxiation, and tissues were harvested after sacrifice. No anesthesia or analgesia was used. 

### Analysis of nephritis, renal immune complexes and renal macrophage infiltration

Kidneys were fixed, paraffin-embedded, sectioned and stained with Periodic Acid-Schiff (PAS) (Sigma-Aldrich, St. Louis, MO) and analyzed by light microscopy. Nephritis was assessed using a semi-quantitative 0 to 4 scale as described previously [5]. 

Renal immune complexes were visualized by immunohistochemistry as described previously [4]. Staining intensity (represented as arbitrary gray units or AGU) of fifty glomeruli per mouse was quantified using region of interest analysis in Axiovision software (v4.6.3.0; Carl Zeiss, Thornwood, NY). 

Renal macrophage infiltration was assessed via immunofluorescence with the macrophage marker Iba1 (Biocare Medical, Concord, CA) as described previously [4,30]. Staining was visualized and quantified using confocal microscopy and Zen 2010 software (v6; Carl Zeiss).

### Analysis of serum immunoglobulin and autoantibody levels

Serum immunoglobulin concentrations were determined by ELISA (Southern Biotech, Birmingham, AL) as described previously [4,5]. The IgG_1_, IgG_2a_, IgG_2b_, and IgG_3_ levels were added together to obtain total serum IgG levels. Serum anti-dsDNA IgG levels were determined by ELISA (Alpha Diagnostics International, San Antonio, TX) as described previously [4,5]. 

### Analysis of markers of tubulointerstitial activation and injury

Renal expression of LCN2 protein was assessed in diluted kidney cell lysates by ELISA (BioPortoDiagnostics, Gentofte, Denmark) according to the manufacturers’ instructions. The protein in the supernatant was quantified using the Bradford method. LCN2 expression levels were normalized to total protein input levels. 

TLR9 protein expression was assessed via immunohistochemical staining with an antibody specific for this receptor (Santa Cruz Biotechnology, Santa Cruz, CA) as described previously [4,19]. Staining intensity (in AGU) of fifty glomeruli per mouse was quantified using Axiovision software. *Tlr9* transcript level was assayed by quantitative RT-PCR. For this analysis, total RNA was isolated from kidney using the Absolutely RNA Miniprep Kit (Agilent Technologies, La Jolla, CA) and cDNA using SS VILO Master Mix (Life Technologies, Carlsbad, CA). PCR was performed using *Tlr9*–specific primers [19], SYBR Green PCR Master Mix (Life Technologies) and the Applied Biosystems 7500 Real-Time PCR System.

### Histological analysis of vasculitis

Spleens were fixed, paraffin-embedded, sectioned and stained with hematoxylin and eosin stain (H&E) (Sigma-Aldrich, St. Louis, MO) and analyzed by light microscopy using sections from age-matched NZW female mice as a healthy control. 

### Analysis of bone quality

Femoral BMD and micro-architectural parameters were measured using Skyscan 1172 micro-CT system (Skyscan, Kontich, Belgium) as described previously [4,31]. Micro-CT scanning parameters were identical to those described previously [4]. Femoral BMD, BV/TV and trabecular number and thickness were quantified with CTAn software (Skyscan).

### Statistical methods

Comparisons were performed using Fishers exact test, Wilcoxon signed-rank test, Mann-Whitney U test, independent or paired samples t-test, or one-way ANOVA with Tukey’s post hoc test where appropriate. Kaplan-Meier survival analysis and log-rank test were used to assess the impact of treatment on lifespan. Statistical analyses were performed using SPSS software (v. 21.0). A two-sided *P* ≤ 0.05 was considered significant. Two-sided *P*-values are provided. Mean ± standard error of the mean is presented.
